# Trait sensitivity to stress and cognitive bias processes in fish: A brief overview

**DOI:** 10.1017/pen.2023.14

**Published:** 2024-01-31

**Authors:** Jhon Buenhombre, Erika Alexandra Daza-Cardona, Daniel Mota-Rojas, Adriana Domínguez-Oliva, Astrid Rivera, Catalina Medrano-Galarza, Paulo de Tarso, María Nelly Cajiao-Pachón, Francisco Vargas, Adriana Pedraza-Toscano, Pêssi Sousa

**Affiliations:** 1 Faculty of Veterinary Medicine, Faculty of Agrarian Science, Animal Welfare Program, Universidad Antonio Nariño, Bogotá, Colombia; 2 Neurophysiology, Behavior and Animal Welfare Assessment, DPAA, Universidad Autónoma Metropolitana, Xochimilco Campus, Mexico City, Mexico; 3 Centro Universitário Mauricio de Nassau, Sobral, Brazil; 4 Especialización en Bienestar Animal y Etología, Fundación Universitaria Agraria de Colombia, Bogotá, Colombia; 5 ICB Biological Sciences, Federal University of Pará, Belém, Brazil

**Keywords:** Fish, personality, stress, neuroscience, cognition, emotion

## Abstract

Like other animals, fish have unique personalities that can affect their cognition and responses to environmental stressors. These individual personality differences are often referred to as “behavioural syndromes” or “stress coping styles” and can include personality traits such as boldness, shyness, aggression, exploration, locomotor activity, and sociability. For example, bolder or proactive fish may be more likely to take risks and present lower hypothalamo–pituitary–adrenal/interrenal axis reactivity as compared to shy or reactive individuals. Likewise, learning and memory differ between fish personalities. Reactive or shy individuals tend to have faster learning and better association recall with aversive stimuli, while proactive or bold individuals tend to learn more quickly when presented with appetitive incentives. However, the influence of personality on cognitive processes other than cognitive achievement in fish has been scarcely explored. Cognitive bias tests have been employed to investigate the interplay between emotion and cognition in both humans and animals. Fish present cognitive bias processes (CBP) in which fish’s interpretation of stimuli could be influenced by its current emotional state and open to environmental modulation. However, no study in fish has explored whether CBP, like in other species, can be interpreted as long-lasting traits and whether other individual characteristics may explain its variation. We hold the perspective that CBP could serve as a vulnerability factor for the onset, persistence, and recurrence of stress-related disorders. Therefore, studying fish’s CBP as a state or trait and its interactions with individual variations may be valuable in future efforts to enhance our understanding of anxiety and stress neurobiology in animal models and humans.

The functional homology of neural regions in fish is well conserved in rodents, and their behavior exhibits sufficient complexity to enable translation to both rodents and humans (Khan & Echevarria, 2017). As such, biological traits that are similar between fish and mammals have been widely utilized in models of anxiety (Egan et al., 2009) and stress neurobiology (Collier et al., 2017; Song et al., 2016, 2018).

To avoid any confusion about the use of the term “stress” in this article, we provide the following brief definitions: The stress response is an organism’s adaptive reaction to restore homeostasis when encountering a threatening stimulus or event (a stressor) (Chrousos, 2009), resulting in either adaptive or maladaptive consequences, known as “eustress” and “distress,” respectively (Koolhaas et al., 2011). This response can be modulated by cognitive appraisals, where the organism evaluates the significance of the stimulus based on stored information in memory (Cerqueira et al., 2017, 2020, Koolhas et al., 2011; as seen in fish). According to this perspective, the stress response involves adaptive assessments and subsequent adjustments, allowing animals to respond effectively to both predictable and unpredictable events – a process known as allostasis (McEwen & Wingfield, 2003; as reviewed in fish by Øverli & Sørensen, 2016). In fish as in other species (Faustino et al., 2015), such as rats (Rygula et al., 2015), dogs (Mendl et al., 2010), lambs (Greiveldinger et al., 2011), fowl (Zimmerman et al., 2011), and bees (Bateson et al., 2011), coping with stress and cognition are closely related processes. For instance, a fish’s appraisal of stimuli, rather than the intrinsic characteristic of the stimuli, can have significant effects on stress responses (Cerqueira et al., 2020, 2021) and related emotion-like (Cerqueira et al., 2017) or affective states.

This article explores “emotion-like” or “affective” states, encompassing descriptors with valence (indicating positivity or negativity, reward or aversion, pleasure or displeasure, among other attributes), intensity (low or high), and duration/persistence (Paul & Mendl, 2018). “Affective” is often used interchangeably with “emotion” or “mood” in animal literature (Kremer et al., 2020) across various species, including mammals (e.g., Mendl & Paul, 2020), birds (e.g., Košťál et al., 2020), fish (Buenhombre et al., 2021; Cerqueira et al., 2017), and invertebrates (Perry & Baciadonna, 2017). These states give rise to a multidimensional response that can be objectively assessed through physiological, neurological, behavioral, and cognitive indicators (Kremer et al., 2020).

Affective states can induce cognitive bias processes (CBP) (Mendl & Paul, 2020). (We provided a glossary of the terms related to CBP in Box 1 and their types in fish are further discussed in section two). Thus, CBP have been used to study the interplay between cognitive and emotional processes in various animal species, including fish (Buenhombre et al., 2022; Espigares et al., 2021; Laubu et al., 2019; Tan et al., 2020a). Emotion-like states also seem to affect sensitivity to reward shifts (SRS) (Burman et al., 2008), which is related to a CBP (Kremer et al., 2020). Two primary sources of variation in CBP have been studied: the living environment and personality traits (Kremer et al., 2021).

Box 1. Glossary

Brief definition, and a reference to further reading where appropriate.

*Cognitive bias processes (CBP):* inclinations to process information in particular ways due to affective states. These cognitive biases include attention, memory, and judgment biases (reviewed by Kremer et al., 2021). For example, people in negative states are more likely to make negative (“pessimistic”) judgments about events or stimuli than people in more positive states (Blanchette & Richards, 2010; Harding et al., 2004).

*Attention bias (AB):* refers to the selective allocation of attention to specific stimuli, studied through attention bias tasks that gauge attention allocation (reviewed by Crump et al., 2018)

*Memory bias (MB)*: involves the influence of an individual’s current emotional state on the nature of their recalled memories (Keen et al., 2014). This bias remains unexplored in fish, and animal studies on this subject have only been conducted in rodents (rats: Burman & Mendl, 2018; mice: Takatsu-Coleman et al., 2013).

*Judgment bias (JB):* also known as the “ambiguous cue interpretation” task (ACI) (Rygula et al., 2013), is the propensity to judge ambiguous cues or situations more or less optimistically (reviewed by Lagisz et al., 2020 for nonpharmacological studies, reviewed by Neville et al., 2020 for pharmacological studies).

*Sensitivity to reward shift (SRS):* also known as “sensitivity to negative and positive feedback” (Noworyta-Sokolowska et al., 2019), is an indicator of affect that more or less relies on cognition and may be viewed as a bias in evaluation and involves sensitivity to rewards and losses influenced by emotional states (Burman et al., 2008).

The living environment significantly influences an animal’s affective states, thereby impacting CBP driven by emotions (e.g., Mendl & Paul, 2020). Interventions aimed at inducing negative affective states (e.g., unpredictable housing, Harding et al., 2004; shaking, Bateson et al., 2011); chronic stress, Rygula et al., 2013) increase the likelihood of exhibiting negative cognitive bias (NCB) (Mendl & Paul, 2020), characterized by a tendency to interpret situations pessimistically (Enkel et al., 2009). For example, exposure to acute or chronic stressors in fish has been linked to anxiety-related behaviors (Buenhombre et al., 2021; Collier et al., 2017; Golla et al., 2020). Additionally, exposure to stressors (Tan, 2017) or non-preferred social stimuli (Laubu et al., 2019) has been associated with NCB. Conversely, interventions aimed at inducing positive affective states, such as environmental enrichment or the use of anxiolytic drugs, often result in a more positive or balanced processing bias, referred to as an “optimistic” response (Bateson, 2016). Buenhombre et al. (2022) observed this effect in fish subjected to various forms of environmental enrichment. Similarly, Laubu et al. (2019) found that exposure to preferred social stimuli in fish results in a positive cognitive bias (PCB). These results underscore the influence of the physical and social environment on fish CBP.

In addition to environmental factors, personality traits could also contribute to variations in CBP. For example, calves (Lecorps et al., 2018) and parrots (Cussen & Mench, 2015), characterized as fearful or neurotic, respectively, have exhibited a more pessimistic cognitive bias while housed under the same conditions. Furthermore, housing and personality may interact to affect CBP, as seen in studies with pigs (Asher et al., 2016), cows (Kremer et al., 2021; Lecorps et al., 2018), and hens (Ross et al., 2019).

A different approach analyses CBP as stable and enduring behavioral traits. Consequently, consecutive assays measuring CBP have been employed in rats (e.g., Enkel et al., 2009; Rygula & Popik, 2016) to categorize individuals into two phenotypic traits: those with a stable PCB, referred to as “optimistic,” and those with a stable NCB, referred to as “pessimistic.” This categorization has played a pivotal role in exploring the idea that CBP could be a trait contributing to the development, persistence, and recurrence of stress-related disorders such as depression and anxiety (Noworyta et al., 2021; Noworyta-Sokolowska et al., 2019).

The above suggests that CBP in some species may incorporate aspects of stable personality traits and more transient affective states, similar to CBP in humans (Kluemper et al., 2009; Rygula et al., 2013). Moreover, depending on an individual’s personality, specific subpopulations of animals may exhibit varying sensitivity to environmental influences on CBP (e.g., Asher et al., 2016; Ross et al., 2019). This hints at a potential link between personality traits, CBP, and stress resilience – the ability to manage potential stressors without significant impacts on normal physiology and behavior (Gesto et al., 2018). While research indicates that fish exhibit personality traits (e.g., Castanheira et al., 2017; Toms & Echevarria, 2014), as further discussed in section three, these interactions have not been thoroughly explored in fish. In this context, our review aims to critically analyze and synthesize the current knowledge regarding CBP and personality in fish. We also aim to explore the potential components of CBP and how they might interact with various personality traits, influencing stress resilience or vulnerability in animals. Also, we highlight the relevance of fish studies as models for aspects of human personality.

## 1.1. CBP and their types in fish

In humans, emotions and moods have been shown to lead to CBP (Mendl & Paul, 2020). Typically, individuals experiencing negative affective states (e.g., anxiety) tend to exhibit heightened attention toward threatening stimuli (e.g., angry facial expressions), demonstrate a greater tendency to recall negative memories, and manifest negative judgments concerning future events or ambiguous stimuli (“pessimism”) when compared to those in more positive states (Barnard et al., 2018). Drawing an analogy, similar CBP patterns have been observed in animals, indicating that affective states can also influence attention, memory, and decision-making across various species (Baciadonna & McElligott, 2015; Bethell, 2015; Raoult et al., 2017; Roelofs et al., 2016), including fish (Buenhombre et al., 2022; Espigares et al., 2021; Laubu et al., 2019; Tan, 2017; Tan et al., 2020a).

Among cognitive measures of affective states in fish, various tasks assessing CBP, attention bias (AB), judgment bias (JB), and sensitivity to reward shift (SRS) (definitions provided in Box 1) have been employed as follows. In AB tasks, animal attention is typically assessed by tracking looking times or recording reaction times in response to specific cues. For instance, in sheep (Lee, C. et al., 2016; Monk et al., 2018) and cattle (Lee, C. et al., 2018), anxiogenic drug administration increased looking time at a hatch (opened to reveal a threatening dog), while anxiolytics decreased it. Although eye movements can be tracked in larvae and adult fish (e.g., Dehmelt et al., 2018), there have been no formal AB studies using looking time tasks in fish. Reaction times can reveal how emotional information distracts individuals during a neutral cognitive task, potentially uncovering attentional biases, such as slower responses to negative stimuli, especially in anxious populations (Cisler & Koster, 2010). However, studies of this nature are scarce in animals (Crump et al., 2018) and remain unexplored in fish. To our knowledge, only one study has examined AB in fish, measuring avoidance responses toward a threatening stimulus as an indicator of animal attention (Tan, 2017). In this experiment, a rotating black strip positioned along the edge of a circular aquarium served as the threatening stimulus. Stressed fish exhibited a higher tendency to position themselves in the inner half of the tank, farther away from the threatening stimulus, in contrast to the control fish. This suggests that fish may demonstrate heightened attention toward novel visual stimuli when exposed to stress-like conditions (Tan, 2017).

JB tasks have been adapted from rodents (Harding et al., 2004) to various species (see Lagisz et al., 2020 for a review), including fish. This experimental approach assesses the expectations of favorable or unfavorable outcomes based on previously acquired cues. During training, subjects learn to respond positively (e.g., approach a location) to a positive stimulus (e.g., position and/or color A) to receive a positive outcome (e.g., food) and negatively (e.g., avoid a location) to a different stimulus (e.g., position and/or color B) to avoid relatively negative outcomes, such as receiving no food (Buenhombre et al., 2022; Laubu et al., 2019) or being chased with a net (Espigares et al., 2021) as observed in fish. Subsequently, ambiguous cues are occasionally introduced to evaluate their anticipation of positive or negative outcomes. The hypothesis is that, similar to humans, negative affective states lead animals to respond to ambiguous cues as if they predict a negative event, and vice versa (for a review and meta-analysis, see Lagisz et al., 2020).

So far, both physical and social conditions have been shown to affect JB in fish. Female convict cichlids assigned to non-preferred partners, which were predicted to elicit negative emotions, exhibited a pessimistic bias (Laubu et al., 2019). In contrast, manipulations predicted to elicit positive emotions, such as constant environmental enrichment in zebrafish (Buenhombre et al., 2022) and staying with a preferred partner in cichlids (Laubu et al., 2019), generated positive JB. Additionally, there is evidence suggesting the involvement of certain key genes in JB. For instance, Espigares et al. (2021) found that telomerase-deficient fish exhibited a more pessimistic response toward ambiguity compared to their wild-type conspecifics.

When it comes to SRS, humans, for instance, tend to exhibit greater sensitivity to potential losses than gains (e.g., Dreher, 2007). Moreover, individuals in a negative affective state often show enhanced sensitivity to loss or failure (e.g., Taylor Tavares et al., 2008). The assessment of animals’ SRS tasks can be conducted through operant conditioning studies designed to investigate successive negative or positive contrast effects, as comprehensively reviewed by Rygula et al. (2018). For example, fish can be trained to swim down a channel to obtain either high or lower value food rewards, with reward values being unexpectedly switched, and the effect of this switch on the time taken to complete the action recorded (Tan et al., 2020b). Sensitivity to reward loss has been demonstrated in various mammals, such as rats housed in unenriched conditions, which typically exhibit indicators of a more negative affective state compared to those in enriched housing (e.g., Burman et al., 2008). Similarly, pharmacological manipulations in rats that boost serotonin neurotransmission have been shown to decrease sensitivity to loss and increase reward sensitivity (Bari et al., 2010). However, in the case of fish, goldfish have been observed to display a downshift in performance with reduced rewards but did not perform worse than controls, indicating no sensitivity to reward loss (e.g., Couvillon & Bitterman, 1985). Similarly, in zebrafish, individuals conditioned to high-value rewards did not change their swimming speed when rewards were downshifted, suggesting no sensitivity to reward loss. Housing type did not affect swim time either (Tan et al., 2020a).

## 1.2. Personality in fish

The concept of personality in humans encompasses enduring behavioral, emotional, and cognitive traits that persist over time and across different situations. However, the definition and measurement of personality can vary depending on the research approach employed. In the context of studying personality in nonhuman species, a trait approach is commonly adopted (Khan & Echevarria, 2017). Research indicates that fish exhibit personality traits (known as consistency in behavior and physiology across time and context and which is characteristic of a certain group of individuals) (Castanheira et al., 2013a; Toms & Echevarria, 2014). Fish researchers frequently employ a cluster of overlapping terms, including “personality traits,” “coping styles,” “behavioural syndromes,” “phenotypic expression,” “behavioural plasticity,” and “individual differences” (e.g., Buenhombre et al., 2021; Conrad et al., 2011; Demin et al., 2019). Currently, studies in fishes have identified personality traits such as boldness, shyness (e.g., Thorbjørnsen et al., 2021), exploration, avoidance, aggressiveness, locomotor activity, and sociability (e.g., Conrad et al., 2011; Khan & Echevarria, 2017; Szopa-Comley et al., 2020).

Fish personality traits, akin to those in humans, are conceptualized as latent axes of variation that underlie observed behaviors, and these traits are often quantified using mathematical models (for a detailed explanation, see Conrad et al., 2011; Prentice et al., 2022; Toms et al., 2010). Specific assays, such as the open-field test, the novel tank test, the emergence test, and the Y-maze, among others (e.g., Buenhombre et al., 2021), are employed to position fish along a continuous dimension defined by two or more axes of interest, such as boldness versus shyness. While some studies use only one of these assays to categorize fish, most employ multiple assays and assess various behaviors over time (e.g., Colchen et al., 2017; Conrad et al., 2011; Toms et al., 2010). This approach involves using correlational and multivariate analyses to establish behavioral clusters representing the underlying behavioral axes. For example, if a fish population exhibits variation in aggressiveness, it implies that certain individuals tend to be more aggressive, leading them to display behaviors such as attacking a mirror stimulus, engaging in increased rivalry displays, or frequently chasing tank mates (Prentice et al., 2022). However, recent findings indicate that there are distinct hormonal and genomic responses in fish when they engage in combat with real conspecific opponents compared to when they confront their own mirror images (Balzarini et al., 2014; Oliveira et al., 2016; Teles & Oliveira, 2016).

Research also suggests that fish exhibit “behavioural syndromes,” essentially personality traits that are correlated with each other (e.g., Conrad et al., 2011; Torgerson-White & Sánchez-Suárez, 2022). For instance, in zebrafish (e.g., Ariyomo & Watt, 2012; Martins & Bhat, 2014), stickleback (e.g., Bell & Sih, 2007), and guppies (e.g., Smith & Blumstein, 2010), a recurring “bold-aggression syndrome” has been observed. This relationship may arise from shared physiological and genetic mechanisms, as well as environmental effects (e.g., Conrad et al., 2011; Prentice et al., 2022). However, it is crucial to recognize that not all studies have established a direct link between boldness and aggression (Way et al., 2015). Additionally, the various approaches to defining and measuring boldness (Toms et al., 2010), along with the context-dependent nature of aggressiveness (Conrad et al., 2011; Dahlbom et al., 2012; Zabegalov et al., 2018), can introduce complexities in interpreting this association. Moreover, besides boldness, aggression is often associated with other traits such as activity and dominance, contributing to a broader “aggression” + behavioral syndrome (Zabegalov et al., 2018).

Furthermore, in fish, the shy-bold dimension is associated with individual variations in both behavioral and physiological responses to stressful stimuli, often referred to as “stress coping styles” (e.g., Castanheira et al., 2017; Thörnqvist et al., 2019; Torgerson-White & Sánchez-Suárez, 2022). These trait variations frequently cluster into two contrasting styles, representing the extremes of a continuous axis. Fish can be characterized as either proactive (engaging in active coping or bold behaviors like “fight-flight”) or reactive (exhibiting passive coping or shy behaviors, often labeled as “non-aggressive”) (e.g., Castanheira et al., 2013b; Saraiva et al., 2018). For example, lines of rainbow trout selected for stress-induced plasma cortisol levels exhibited correlated changes in social, feeding, and locomotor behavior (as reviewed by (Øverli et al., 2005). Similarly, wild-type guppies demonstrated evidence of genetic correlation structures between stress-related behavioral traits (such as thigmotaxis and freezing) expressed in open-field trials (OFTs) and the levels of free circulating cortisol produced in response to an isolation and confinement stressor (Houslay et al., 2022).

However, the stress coping style model presents certain challenges. Traits vary along two independent axes: a qualitative coping style axis and a quantitative stress reactivity axis (Koolhaas et al., 2010), which can make it challenging and subjective at times to determine how observed data align with these axes (Houslay et al., 2022). Furthermore, only a limited number of studies in fish have incorporated repeated observations of both endocrine and behavioral stress response traits (Boulton et al., 2015; Thörnqvist et al., 2019), and some of these studies present inconsistent (Boulton et al., 2015) and context-dependent findings (Alfonso et al., 2019; Thomson et al., 2012), complicating the interpretation somewhat (Prentice et al., 2022). Additionally, a recent study suggests that a single divergent stress coping style may not fully capture the diverse range of behavioral clusters beyond the original bimodal reactive–proactive characterization (Rajput et al., 2022). Furthermore, behavioral clusters can be influenced by factors such as social context (Magnhagen & Bunnefeld, 2009), strain, and sex (Rajput et al., 2022; Wong et al., 2019).

## 1.3. Trait sensitivity to stress and cognitive bias processes

From fish through humans, some individuals are likely better at coping with adverse conditions than others (Sørensen et al., 2013). Experience (e.g., habitat complexity and rearing conditions) (Lee, C. J. et al., 2018) and genetic factors (e.g., fibroblast growth factor receptor 1a simultaneously increase aggression, boldness, and exploration in adult zebrafish) (Norton et al., 2011) may control this among individual differences in fish’s stress responses (Sørensen et al., 2013). These consistent differences among fishes modulate the way they perceive and react to their environment, which in turn affects their robustness (Vindas et al., 2017) and resilience (Buenhombre et al., 2021) to challenges. For instance, proactive individuals create routines, are explorative and risk-taking (Sih et al., 2004), and seem to have a high level of active avoidance, locomotor activity, and low flexibility in behavioral responses when faced with challenges, while reactive individuals behave with the opposite patterns (Ruiz-Gomez et al., 2011; Sih et al., 2004). In addition, proactive individuals exhibit typical physiological and neuroendocrine characteristics such as higher expression of dopamine and opioid receptors (Thörnqvist et al., 2019), lower levels of 5-hydroxyindoleacetic acid (5-HIAA) and baseline ratio of 5-HIAA/serotonin (5-HT) (Øverli et al., 2001; Winberg & Thörnqvist, 2016), lower hypothalamus–pituitary–adrenal/interrenal activity (Øverli et al., 2007; Silva et al., 2010) as compared to reactive individuals.

Regarding individual differences in cognition, these have rarely been addressed in fish compared with humans and rodents (Lucon-Xiccato & Bisazza, 2017). Some studies suggest that fish’s personality traits can exert different influences on cognitive performance, depending on the specific task. Faster learning rates to avoid experiencing an unpleasant stimulus (an aversion learning paradigm that requires avoidance or reduced levels of activity) have been observed in risk-averse reactive individuals (Baker & Wong, 2019; Budaev & Zhuikov, 1998), and it has been hypothesized that reactive individuals may perceive stressors as more threatening, which could facilitate faster encoding of aversive experiences (Baker & Wong, 2019). The expression of two neural plasticity and neurotransmission-related genes (npas4a and gabbr1a) may be involved in fear learning differences among stress coping styles (Baker & Wong, 2021). On the contrary, the more risk-prone proactive individuals tend to show faster acquisition of memories that require higher levels of activity or paradigms with positive and rewarding valence (Baker & Wong, 2019; Lucon-Xiccato & Bisazza, 2017). Likewise, Ferrari et al. (2010) found that shy rainbow trout had better memory for a predator odor 8 days after conditioning it with alarm cues from conspecific skin. The latency of the fish to emerge from an opaque chamber placed in a novel tank after a 20-min habituation was used to categorize the trout. The longer the latency to emerge, the shier the individual was. However, it is worth noting that not all studies have established these associations (Kareklas et al., 2018; Vital & Martins, 2013).

Despite several studies exploring numeracy, spatial cognition, social cognition (as reviewed by Lucon-Xiccato & Bisazza, 2017; Salena et al., 2021), and more recently, some studies exploring CBP in fish, the influence of fish’s personality traits on cognitive processes other than cognitive achievement has not been explored yet. Nonetheless, fish personality traits could likely be intertwined with CBP, as observed in pigs (Asher et al., 2016), cows (Kremer et al., 2021), hens (Ross et al., 2019), and dogs (Barnard et al., 2018).

CBP in animals is often considered a transient state influenced by the animal’s mood (Mendl et al., 2009). However, an alternative perspective has emerged, suggesting that CBP could also be seen as enduring traits (Faustino et al., 2015). Evidence from studies in rodents (e.g., Noworyta et al., 2021; Noworyta-Sokolowska et al., 2019; Rygula et al., 2013) and calves (Lecorps et al., 2018) supports this idea, demonstrating that these animals exhibit stable individual differences in their levels of pessimism or optimism within CBP. Furthermore, Rygula et al. (2013) found that rodents categorized as optimistic or pessimistic after chronic stress exposure consistently made pessimistic judgments about ambiguous stimuli. This suggests that CBP in animals, similar to humans, may encompass characteristics of both a trait and a transient state (Kluemper et al., 2009; Rygula et al., 2013).

CBP has also been considered a vulnerability factor for the etiology, maintenance, and recurrence of stress-related disorders (e.g., Clark & Beck, 2010). In humans, patients with these disorders often exhibit NCB (e.g., Disner et al., 2011; Robinson, 2019). Similarly, in rodents, environmental, pharmacological, and genetic manipulations that induce stress-like states (reviewed by Nguyen et al., 2020) have been found to cause NCB. Furthermore, rats classified as pessimistic tend to show higher vulnerability to stress-induced anhedonia (Rygula et al., 2013), increased sensitivity to reward losses (Rygula & Popik, 2016), and an inflammatory immune profile compared to optimistic animals (Curzytek et al., 2018).

In fish, Espigares et al. (2021) observed NCB in a mutant strain of zebrafish with shorter telomeres and in aging fish with age-related telomere shortening. These mutant zebrafish exhibit early phenotypic alterations, including increased inflammation, which are common in aged organisms and may contribute to the NCB observed in the telomerase-deficient mutants. Additionally, Espigares et al. (2022) found that fish categorized as pessimistic increase their reproductive investment after chronic stress, leading to increased vitellogenesis. These findings suggest that in animals, including fish, optimistic and pessimistic traits and states may confer different levels of resilience to individuals in stressful situations (Faustino et al., 2015), indicating that pessimistic traits/states may be less resilient to stress, and vice versa.

## 2. Conclusions

Clearly, zebrafish models of CBP are still in the early stages of development, and numerous unanswered questions persist (Table 1). For instance, although zebrafish do exhibit CBP, these responses may be influenced by individual differences, such as age, sex, personality, and strain, owing to environmental and genetic variations, along with their interplay (Volgin et al., 2019). Consequently, CBP research in fish must meticulously consider, distinguish, and control for these factors and their interactions, with particular attention to personality traits. The intricate nature of these variables can potentially complicate the interpretation of CBP results, as we have discussed in this review across various species. Moreover, CBP may encompass both transient affective states and more enduring personality traits (e.g., Faustino et al., 2015; Kluemper et al., 2009; Rygula & Popik, 2016). Consequently, CBP in fish could serve as a valuable model for disentangling which of these components is being assessed and how negative or positive states/traits influence an animal’s evaluation of ambiguous stimuli. This understanding can aid in identifying individuals who may exhibit higher stress resilience. For example, rodents in negative states have been shown to be less resilient to aversive events, as demonstrated by Rygula et al. (2013), and pessimistic fish display different reproductive outcomes after experiencing stress, as observed by Espigares et al. (2022). Additional research is warranted to establish the validity and reproducibility of CBP as suitable measures of affective states or traits in fish. Finally, insights gained from fish research on CBP may contribute to cognitive models suggesting that stress-related disorders in humans are linked to biases in cognitive processing (Beck, 2008).

**Table 1.** Selected outstanding questions in fish CBP research

Can CBP be considered enduring traits, emotional states, or a combination of both?
Are there stable individual differences in baseline CBP?
How long do affective states influence CBP?
Do CBP differ between laboratory strains, wild-derived, and wild-caught zebrafish?
Do CBP vary by sex?
How does aging affect CBP?
Do the physical conditions of the organism interact with CBP?
Do social interactions affect CBP?
Can pharmacological interventions modulate CBP?
Are negative and positive housing and social contrasts effective models for affect manipulation and CBP?
Are negative or positive states and traits associated with stress vulnerability or resilience?
Are changes in CBP related to physiological dysregulation markers?
Do personality traits like shyness and boldness influence CBP states or traits?
Do fish exhibit memory bias?
Are fish sensitive to reward shifts?
Do fish show modulation in AB in response to stimuli assumed to induce positive affect?
How does the experimental setup (cues, trial duration, training sessions, tasks, etc.) affect trait and state CBP variations?
Does an animal’s prior experience with CBP tests influence responses to subsequent tests?
How does domestication influence CBP in various fish species and strains?
How have ecological pressures shaped CBP?
Which neuronal areas are involved in CBP?
What neural mechanisms and circuits underlie CBP?
Are neurotransmitters correlated with differences in NCB and PCB?
How do negative or positive states and traits affect an animal’s evaluation of ambiguous stimuli?
Can distinct genes differentially modulate CBP?
What are potential epigenetic mechanisms of CBP?
Can microbiota affect CBP?
