# Trait sensitivity to stress and cognitive bias processes in fish: A brief overview – ERRATUM

**DOI:** 10.1017/pen.2025.1

**Published:** 2025-02-20

**Authors:** Jhon Buenhombre, Erika Alexandra Daza-Cardona, Daniel Mota-Rojas, Adriana Domínguez-Oliva, Astrid Rivera, Catalina Medrano-Galarza, Paulo De Tarso Teles Dourado De Arago, María Nelly Cajiao-Pachón, Francisco Vargas, Adriana Pedraza-Toscano, Pêssi Sousa

This article was originally published with an error in one of the author’s name, Paulo de Tarso. The correct spelling is Paulo De Tarso Teles Dourado De Arago. The publisher apologises for this error, and this has been corrected.

